# Are all helicopter dispatches really necessary? a cross-sectional study

**DOI:** 10.5249/jivr.v15i1.1778

**Published:** 2023-01

**Authors:** Nina Farzan, Seyed Mohammad Hashem Montazeri, Ashkan Beiranvand, Seyed Mojtaba Alavi, Mostafa Vahedian

**Affiliations:** ^*a*^ Clinical Research Development Center, Qom University of Medical Science, Qom, Iran.; ^*b*^ Student Research Committee, School of Medicine, Qom University of Medical Sciences, Qom, Iran.; ^*c*^ Department of Biostatistics and Epidemiology, Neuroscience Research Center, School of Medicine, Qom University of Medical Science, Qom, Iran.

**Keywords:** Air Ambulance, Helicopter EMS, Helicopter Emergency Medical Services, HEMS, Helicopter Air Ambulance

## Abstract

**Background:**

Pre-hospital emergency care is a critical part of the health care system. Helicopter emergency medical service (HEMS) is a novel part of the medical services of the health care delivery system. The goal of these medical services is to provide appropriate treatments at the right place and time. The pre-hospital emergency is the first line of providing emergency care to patients and injured. To reduce the death and disability of patients, the optimal performance of various pre-hospital emergency branches, such as HEMS, is needed. Thereby, it is essential to pay attention to the importance of hospital wards and patient transfer. However, the HEMS can impose a high cost on the health care system. Due to a lack of evidence, in this study, we will investigate the reasons and consequences of transferring patients by HEMS in Shahid Beheshti Hospital in Qom.

**Methods:**

This study is a cross-sectional descriptive study. In this study, sampling was done by the census, and all patients were transferred by air ambulance during the year 1400 (March 2021 to 2022) using the emergency department of Qom province, and the study of patient health records was performed.

**Results:**

A significant percentage of patients (46.8%) were discharged from the emergency department in the first 6 hours. Most of the patients (79%) did not need surgery. 41.1% of the patients were traumatic patients with no pathological findings in their imaging.

**Conclusions:**

many cases did not necessarily need HEMS to transfer. It is recommended to choose the candidates more carefully for air ambulance transfer to reduce unnecessary costs.

## Introduction

A Pre-hospital emergency care includes a critical part of the primary care in healthcare systems, performed before the patient arrives at the hospital. It can differ based on the type of incident, such as trauma, medical illness, and the characteristics and degree of vulnerability of the patient. Accident scene management, proper and quick transfer of the patients, performing vital medical procedures, etc., can reduce the mortality and disability of the patients.^1^ Helicopter emergency medical services (HEMS) is an efficient branch of emergency pre-hospital care. The first aeromedical transportation of patients dates back to the 1800s, the Franco-Prussian war.^2^

Helicopters can provide fair and efficient access to vital medical services for patients, especially in a wide geographical area.^3^ Mostly, the outcomes of patients transported by air ambulance are better in such cases, as myocardial infarction, stroke, and trauma. It can transport several patients at the same time. On the other hand, medical contraindications such as active labor, untreated and severe pneumothorax, the aggressiveness of the patient, and even the high weight of the patient should be taken into account in addition to bad weather conditions. These are considered the limitations of the use of HEMS.^4,5^ Despite the advantages of the air ambulance, no one can ignore the high cost of flights and technical services, and emergency crew of an air ambulance. Some steps were taken to lower the costs of HEMS in the US.^6^ The main reason for the use of air ambulances in Iran was trauma cases. According to previous studies, despite being expensive, it has been able to play a helpful role in reducing the period of hospitalization and the death of patients.^5,7^

Due to the importance of HEMS and the lack of studies on this issue, especially in developing countries like Iran, we tried to investigate the reasons for using this type of emergency, the exact characteristics of the patients, and the patient outcomes who referred to Beheshti Hospital, Qom. We also tried to evaluate the efficiency and necessity of the HEMS in Iran.

**Patients and Methods:**

**Study design and setting**

This cross-sectional study was done from March 2021 to March 2022 at Shahid Beheshti hospital complex in Qom, Iran. We did not interfere with the patient’s diagnostic process or discharge from the hospital. We just observed the outcomes of all patients who were referred to the emergency department by air ambulance.

**Outcomes and measurements**

A checklist was designed to collect the patients' data, including demographic features (age, gender), mechanism of the event (accident, cardiopulmonary, trauma/falling, obstetrics and gynecology, poisoning, loss of consciousness, gunshots, burns, bites, and strokes), diagnosis, triage level, admission section (intensive care unit, general ward or discharge after initial management in less than 6 hours), and outcomes (death or discharge).

**Aims**

The main goal of this study is to determine the reasons and consequences of the patients who transferred to the hospital by air ambulance regarding the demographic characteristics, diagnosis, and other mentioned variables.

**Ethical issues**

The Medical Ethics Committee of Qom University of Medical Sciences approved and supervised all stages of the study according to the Helsinki ethics criteria (ethical code: IR.MUQ.REC.1401.011). We confirm that all experiments and methods were performed under relevant guidelines.

**Statistical analysis**

Data were entered into the statistical software SPSS 23 (SPSS Inc, Chicago, IL) and analyzed using descriptive statistics. Qualitative data were expressed as frequencies (percentages), and quantitative data were described with statistics such as the mean and the standard deviation. Finally, the results were presented with the aid of descriptive graphs and tables. The Chi-2 test and Fisher's exact test were used for data analysis. A significant level of less than 5% is considered.

## Results

One hundred twenty-four patients transferred by HEMS to Shahid Beheshti Hospital, Qom, were studied. The minimum age of the transferred patient was one year old, and the maximum was 83. The average age of people was 32.85, the mean age of people was 32, and the standard deviation was 18.184. The average triage level of the patients was 1.87, the median triage level was two, and the standard deviation was 0.710. The lowest number at the triage level was one, and the highest was 3. The highest frequency distribution of patients was related to the age range of 20 to 40 years. The highest frequency distribution of triage levels of patients was related to triage level 2. Eighty-three cases (66.9%) were male, and forty-one of them were females (33.1%). Fifty-eight patients (46.8%) were discharged from the emergency department in the first 6 hours, and thirty-one patients (25%) were admitted to the general ward. Twenty subjects (16.1%) were admitted to the intensive care unit (ICU), and just three patients (2.4%) died in the emergency room. Ten patients (8.1%) left the hospital against medical advice by their responsibility in the emergency room. One of the patients (0.8%) absconded from the emergency room, and one case was transferred to another hospital. 98 patients (79%) did not undergo surgery, twenty-five patients (20.2%) underwent surgery and a patient (0.8%) underwent angiography.

Most patients (91.1%) were referred to the hospital due to vehicle accidents. 4% due to falling from a height, and 2.4 % due to carbon monoxide poisoning. Other less common causes were gas cylinder explosion (0.8%), chest pain (0.8%), and fighting (0.8%). Overall, 101 patients (81.5%) were discharged alive, and just five (4%) expired. 

Most of the transferred patients did not undergo surgery, and among the patients who underwent surgery, most of the surgeries were related to emergency thoracic surgeries. (Table 1)

**Table 1:** Frequency and percentage of surgery types among patients.

Surgery type	Frequency (number)	percentage
No need for surgery	97	78.2
Non-emergent orthopedic surgery	6	4.8
Emergent neurosurgery	2	1.6
Non-emergent neurosurgery	2	1.6
Emergent laparotomy	2	1.6
Emergent thoracic surgery	8	6.5
Emergent multi-organ surgery	3	2.4
Non-emergent multi-organ surgery	1	0.8
Percutaneous coronary intervention	1	0.8
Non-emergent reconstructive surgery	2	1.6

According to the final diagnoses of the patients, the majority of the transferred patients (41.1%) were traumatic patients with no pathological imaging findings. The second most common patients were traumatic patients who did not require surgery. Carbon monoxide poisoning and transmission of a pregnant woman were the lowest diagnoses of the patients. (Table 2 )

**Table 2:** Frequency of patients based on final diagnosis at the hospital after evaluations.

Diagnosis	Frequency (number)	percentage
Trauma with normal imaging findings	51	41.1
left the hospital against medical advice by their own responsibility (before the final diagnosis)	9	7.3
Myocardial infarction	1	0.8
patient died before admission	3	2.4
Extensive lesion of the nasal area	1	0.8
Head trauma requiring emergent surgery	3	2.4
Upper limb trauma requiring non-emergent surgery	4	3.2
Lower limb trauma requiring non-emergent surgery	1	0.8
Chest trauma requiring emergent surgery	3	2.4
Multi-organ trauma requiring emergent surgery	9	7.3
Multi-organ trauma requiring non-emergent surgery	4	3.2
Non-emergent surgical facial trauma	2	1.6
Vertebral trauma requiring non-emergency surgery	3	2.4
Head trauma without surgery	11	8.9
Non-surgical multiple organ trauma	3	2.4
Non-surgical limb trauma	12	9.7
Carbon monoxide poisoning	3	2.4
Pregnant woman with trauma	1	0.8

## Discussion

The goal of this study was to investigate the reasons and consequences of transferring patients by HEMS to Beheshti hospital. This study model seems necessary due to the high financial cost and the required specialized staff for the air ambulance. 

We found that most of the patients were young men, and less than half of the patients were so well that they were discharged from the hospital after initial examinations (6 hours). In general, most referred patients had a good prognosis, and death has occurred in a few cases.

Also, the main reason for the use of air ambulances in Iran was due to road traffic injuries. The most common diagnosis of the patients was trauma, and in half of the cases, all imaging findings were normal. Only about one-fifth of the patients needed surgery. Half of these surgeries were not critical and emergent.

According to Iran`s HEMS report in 2014, about 77% of HEMS missions during recent years were allocated to road accidents. In the statement of HEMS in 2017, about 56% of the patients of this service were from road traffic injuries. Our data is broadly consistent with these reports.

There is a lack of studies regarding the consequences of patients transferred by HEMS and dispatch criteria in Iran. Moradian et al. investigated the reasons and consequences of transporting patients by air ambulance to Shiraz hospitals. They showed that 68% of patients transported by air ambulance were men, and 32% were women. 43% were in the age group of 30 to 60 years. 50.5% of the patients were admitted to the general ward, 29% were admitted to the ICU, 9% underwent surgery, and 4.7% died in the hospital. The most common cause of the transfer of patients was accidents and trauma.^3,8^ According to a study, patients transferred by air ambulance had more critical situations compared to patients transferred by land emergency.^8^ Based on their data, most of these patients had severe injuries to the neck, chest, abdomen, and back. The outcomes of these patients were not determined. These studies and our study suggest more supervision is needed for helicopter dispatches because there are limited resources with high-cost demand.

Chang et al. conducted a retrospective cohort study to predict the clinical outcome of patients transported by air ambulance. Three hundred seventy cases were studied with an average age of 54.5 years. 71.6% of the patients were male. The average transfer time of patients was 1.4 hours. The 7-day, 30-day, and hospital mortality rates were 10.3%, 14.1%, and 14.9%, respectively. Among the 370 individuals, 32.5% had neurological problems, 24.9% had heart problems, and 16.2% were referred due to trauma. The factors related to 7-day mortality were age, GCS, and hematocrit level. The age, GCS, hematocrit level, hemodynamic instability, and tracheal intubation were related to 30-day mortality. Mortality did not have a relationship with the type of transportation, flight length, rescue crew, transportation cause, and clinical factors of the patient.^9^

Parnia et al. evaluated the critically ill patients transported by HESE in cruise ship departments. One hundred four patients were included in this study, 65 of them were men, and 39 of them were women, with an average age of 68.7. 80.8% of their patients were internal and cardiac cases and 19.2% were surgical cases. 96.1% of air transports were necessary. Complications and mortality during the transfer were 8.5% and 2.9%, respectively, and most were related to cardiac causes. Ninety-eight patients were hospitalized, 10 cases got new complications, and five individuals died. At the end of this study, it showed that the HEMS has an influential role in transporting patients.^10^ According to two studies, we can find out that, unlike in other countries, the etiology of air ambulance use is different in Iran. Some studies have demonstrated that HEMS is not cost-effective and useless for some patients.^11^

Our study limitations are that we examined only one air base and hospital, and perhaps generalizing these results to the entire country requires conducting more extensive and multicenter studies. We have not evaluated the mental characteristics of patients and the severity of the accident. Maybe other variables alter the decision to use an air ambulance. According to this study and other Iranian studies compared to other countries, it can be concluded that there is a need to modify and formulate new guidelines for dispatching a helicopter.

Further studies with a more extensive statistical community are needed to generalize the findings.

## Conclusion

Most of the patients transported by helicopter were traumatic events with normal imaging. Increasing public awareness of the medical system and increasing self-care and prevention of traumatic events can reduce emergency cases. A significant percentage of patients were discharged from the emergency department in the first 6 hours, and most transferred patients did not need emergent surgery. Based on these findings, we need a fundamental revision of the HEMS guidelines to impose a less financial burden on the health care system.
